# Aseptic lysis L2–L3 as complication of abdominal aortic aneurysm repair

**DOI:** 10.1007/s10195-014-0308-9

**Published:** 2014-07-15

**Authors:** Federico Mancini, Andrea Ascoli-Marchetti, Luca Garro, Roberto Caterini

**Affiliations:** 1 Department of Orthopaedics and Traumatology, University of Rome “Tor Vergata”, Viale Oxford, 81, 00133 Rome, Italy; 2 Department of Vascular Surgery, University of Rome “Tor Vergata”, Rome, Italy

**Keywords:** Contained aneurysm rupture, Vertebral erosion, False aneurysm, Endovascular repair

## Abstract

Osteolytic vertebral erosion is usually related to tumours, spondylitis or spondylodiscitis. Few reports in the literature describe lytic lesions of anterior lumbar vertebral bodies resulting from abdominal aortic aneurysm or false aneurysm. We report a case of abdominal aortic false aneurysm that caused lytic lesions of the second and third vertebral bodies in an 80-year-old man who underwent endovascular aneurysm repair. Fluoroscopy guided biopsy excluded infection or tumour. We performed a posterior spinal fusion and decompression because of bone loss of the second and third lumbar vertebral bodies and central stenosis. Postoperatively the patient showed satisfactory relief in low-back and thigh pain but, unfortunately, he died 1 month after surgery because of respiratory complications. This case suggests that when a lytic lesion of a lumbar vertebral body is discovered in a patient who has undergone endovascular aneurysm repair, an abdominal aortic false aneurysm may be the cause of the vertebral erosion even in cases without infective pathogenesis.

## Introduction

Osteolytic vertebral erosion is usually related to tumour, spondylitis or spondylodiscitis. Vertebral erosion determined by an abdominal aortic aneurysm is rare but already described [1–5]. Very few cases of vertebral lesion caused by false aneurysm secondary to prosthetic stent have been reported [5–9], and this is the first case of vertebral erosion due to a false aneurysm in a patient who underwent endovascular aneurysm surgery in absence of disco-vertebral infection.

## Case report

**Fig. 1:**
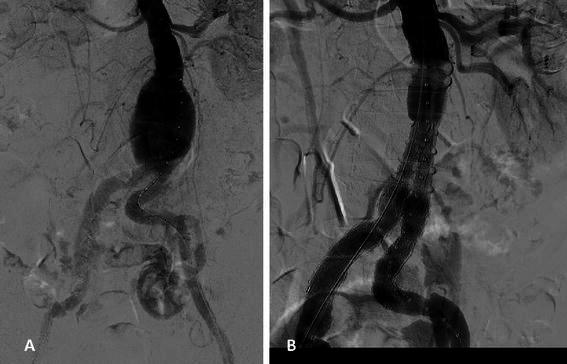
Intraoperative angiography before (**a**) and after (**b**) the endoprosthesis deployment. No signs of rupture are evident

**Fig. 2:**
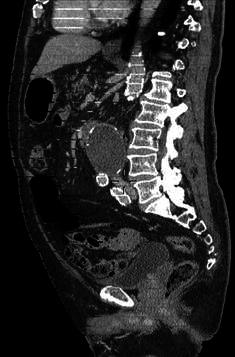
Computed tomography scan 1 month after endovascular abdominal aortic aneurysm repair. No signs of vertebral erosion are present

**Fig. 3:**
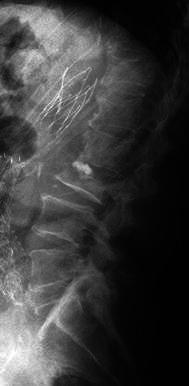
Lumbar spine X-ray showing vertebroplasty procedure performed 3 months after aneurysm repair

**Fig. 4:**
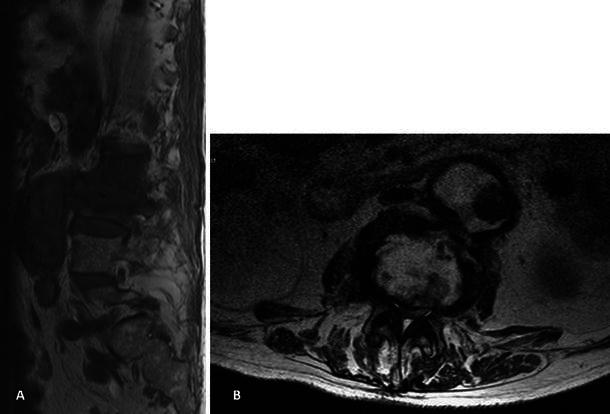
**a** MRI 9 months after endovascular abdominal aortic aneurysm repair showed the false aneurysm and its relation to L2–L3 vertebrae. **b** L2–L3 central stenosis

**Fig. 5:**
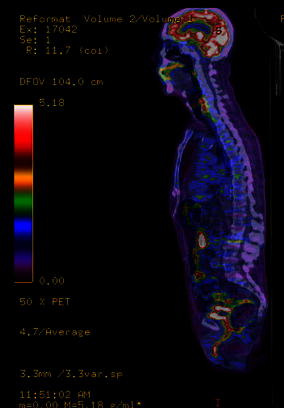
PET-CT section 9 months after endovascular abdominal aortic aneurysm repair was not significative of infection

**Fig. 6:**
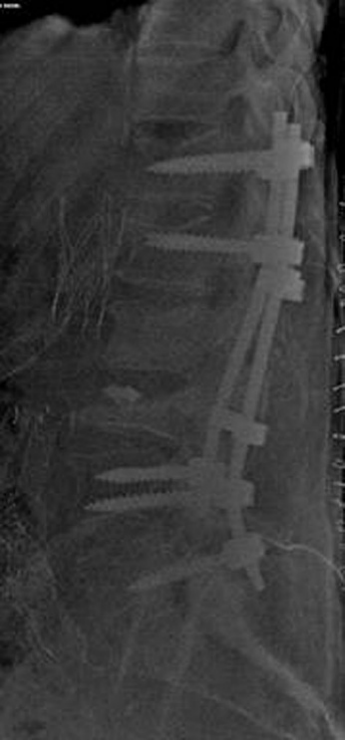
Postoperative X-ray of lumbar spine

An 80-year-old man, with multiple co-morbidities was admitted to the author’s hospital for severe low-back pain, lower limbs motor impairment and bilateral thigh pain. Nine months before admission to our department, he underwent endovascular aneurysm repair (EVAR) for an abdominal aortic aneurysm (Fig. 1). A computed tomography (CT) scan performed 1 month after the endovascular abdominal aortic aneurysm repair did not show any signs of lumbar vertebral or disc erosion (Fig. 2). Three months after the endovascular procedure, he developed a progressive lower back pain and bilateral thigh pain that did not respond to conservative treatments. At this time, in another hospital, MRI of the lumbar spine showed severe bone loss in the anterior half of the third lumbar vertebral body and L3 vertebroplasty was performed without any significant relief of the symptoms (Fig. 3). On admission in our hospital, 9 months after EVAR, clinical evaluation showed severe low-back pain, bilateral thigh pain, motor deficit of the lower limbs and the patient was not able to walk (Frankel C) [10]. Laboratory findings were within normal limits: haemoglobin, 12.5 g/dl (normal 12–16 g/dl), white blood cell count, 6.3 × 10^9^/l (normal 4.3–10.8 × 10^9^/l) with a normal differential, erythrocyte sedimentation rate 25 mm/h (normal 2–30 mm/h), C-reactive protein 2.4 mg/l (normal 0.00–3.00 mg/l). A new MRI of the lumbar spine, performed at admission, showed the false aneurysm and its relation to L2–L3 bodies causing vertebral and disc erosion (Fig. 4). Fluoroscopy guided biopsy was performed, but it was negative as regards microbiological and histopathological examination for tumours or infections. A more accurate evaluation of MRI of the lumbar spine uncovered an extensive abdominal aortic false aneurysm, corresponding with the prosthetic stents that had eroded the vertebral bodies of L2–L3. The vascular surgeon did not consider revision surgery necessary at that time. Laboratory findings, fluoroscopy guided biopsy and PET-CT (Fig. 5) had excluded infection, and for this reason we avoided draining the fluid at the L2–L3 disc space, but we opted for a posterior decompression of the central canal stenosis, between L2 and L3, to improve the neurologic condition of the patient. In addition a long and extensive instrumentation of T12–L5 was performed to avoid the risk of implant failure due to the presence of severe vertebral osteopenia (Fig. 6). Moreover, an autologous iliac bone graft was utilized to obtain better postero-lateral fusion. In the disc space L2–L3 we just found abundant serum-hematic fluid. Another biopsy of the L2–L3 disc performed during surgery confirmed the absence of tumours or infections. Postoperatively, laboratory findings were: haemoglobin 9.2 g/dl, white blood cell count 8.5 × 10^9^/l, erythrocyte sedimentation rate 35 mm/h, C-reactive protein 3.00 mg/l. After surgery the patient obtained excellent relief of low-back and thigh pain with a satisfactory regain of walking (Frankel D). Unfortunately, 1 month after surgery the patient died because of respiratory complications. We excluded sepsis as a possible cause of death because the patient in the postoperative period had no fever and laboratory findings were within normal limits.

## Discussion

Osteolytic vertebral erosion is usually caused by neoplasms, spondylitis or spondylodiscitis. Some reports in the literature report that an abdominal aortic aneurysm can cause erosion of the lumbar vertebral body, due to a progressive aneurysmatic sac expansion [1–5]. Few authors [6, 7] have reported lumbar vertebral erosion resulting from abdominal aortic contained rupture aneurysm in patients surgically treated for an abdominal aortic aneurysm by a conventional open surgical repair. Other authors have reported vertebral lesions resulting from endovascular abdominal aneurysm repair complicated by an infection [8, 9]. To the best of our knowledge, this is the first report of a case in which an endovascular aneurysm repair (EVAR) for an abdominal aortic aneurysm was complicated by an abdominal aortic false aneurysm which caused severe erosion of two lumbar vertebral bodies and disc through an inflammatory mechanism, without signs of infective pathogenesis. It is possible to assume that, despite the endovascular procedure, the pseudo-aneurysmatic sac can cause an inflammatory stimulus that is erosive for the adjacent vertebrae and discs. Pre-existing osteopenia, frequently observed in old patients, can contribute to the development of the vertebral erosion.

If the patient is in good general condition, an anterior approach with the removal of the prosthesis [11] and L2–L3 decompression and fusion should be considered.

We assume that when a lytic lesion of a lumbar vertebral body or disc is discovered in a patient treated for an abdominal aortic aneurysm by endovascular repair, an abdominal aortic false aneurysm can be the cause of the vertebral or disc erosion even in cases without infective complication.
